# Genetic Polymorphism of *agr* Locus and Antibiotic Resistance of *Staphylococcus aureus *at two hospitals in Pakistan

**DOI:** 10.12669/pjms.301.4124

**Published:** 2014

**Authors:** Sadia Khan, Faisal Rasheed, Rabaab Zahra

**Affiliations:** 1 Sadia Khan, M. Phil, Department of Microbiology, Quaid-i-Azam University, Islamabad 45320, Pakistan.; 2 Faisal Rasheed, M. Phil, Department of Microbiology, Quaid-i-Azam University, Islamabad 45320, Pakistan.; 3 Rabaab Zahra, PhD, Department of Microbiology, Quaid-i-Azam University, Islamabad 45320, Pakistan.

**Keywords:** *S. aureus*, * agr*, MRSA, MSSA

## Abstract

***Objective: ***The accessory gene regulator (*agr*) locus in *Staphylococcus aureus* (*S. aureus*) is a global regulator of quorum sensing and controls the production of virulence factors. This study was carried out to investigate the *agr* specific groups both in methicillin resistant and sensitive *Staphylococcus aureus* (MRSA and MSSA) and their relation with antibiotic resistance.

***Methods: ***A total of 90 clinical *S. aureus* isolates were studied from two tertiary care hospitals. The isolates were identified by standard biochemical tests. Methicillin resistance was confirmed by oxacillin and cefoxitin resistance. Multiplex PCR was used to determine the *agr* groups.

***Results: ***MRSA prevalence was found to be 53.3%.The *agr* groups’ distribution in MRSA was as follows: 22 (45.8%) belonged to group I, 14 (29.1%) belonged to group III and 2 (4.1%) belonged to group II. *agr*IV was not detected in MRSA. For 17 isolates, the *agr *group was not detected.*agr* III isolates showed higher antibiotic resistance than *agr*I isolates except in case of oxacillin and linezolid.

***Conclusions: ***Strict infection control policy and antibiotic guidelines should be adopted to control the problem of MRSA. Higher prevalence of *agr* I and *agr* III shows that they are dominant *agr *groups of our area.

## INTRODUCTION

*Staphylococcus aureus *is an extracellular Gram positive pathogen, which causes a number of infections such as pneumonia, endocarditis, and septic arthritis.^1^ In many cases, the infection originates from hospital derived antibiotic resistant bacteria, among which the most common is MRSA whose prevalence varies markedly between different regions and hospitals.^2^ Within hospitals, MRSA accounts for 40–70% of infections in Intensive care units^3^ and overall it is responsible for 50% or more of hospital acquired infections in many countries. Normal nasal carriage of *S. aureus *is 25-30% whereas less than 2% of normal individuals are colonized with MRSA.^4^

In Pakistan, the prevalence of MRSA has increased tremendously over the years. It was reported as 5% in 1989 and since then has increased up to 51%. It is reported to range from 42 to 51%, increasing from the 1990 to 2000^5^^,^^6^ and from 19.5% in 2001 to 40% in 2008.^7^

*S. aureus *produces many virulence factors comprising of toxins and enzymes, regulated by *agr *and *sar*systems.^8^ The *accessory gene regulatory (agr) *system down regulates the expression of surface proteins while up regulates the expression of expoproteins. It encodes two transcripts, RNAII and RNAIII where RNAII encodes for *agrA*, *agrB*, *agrC *and *agrD*. *S*. *aureus *isolates can be divided into four *agr *groups on the basis of the specificity of the auto-inducing peptide (AgrC).^9^ Further, *S. aureus *strains belong to specific *agr*groups^10^ implicating the importance of the knowledge of *agr *gene groups.

The current study was designed to analyze the genetic polymorphism of *agr *locus among *S. aureus* isolates and to assess its relationship with antibiotic resistance profile.

## METHODS

***Bacterial Isolates: ***The study includes a total of 90 *S. aureus *clinical isolates, out of which 35 were collected from Holy Family Hospital, Rawalpindi whereas, 55 isolates were collected from Microbiology Laboratory, Pakistan Institute of Medical Sciences, Islamabad during the months of April to Oct 2011. These isolates were taken from different sources where 18 were from nasal swab, 50 from pus, 3 from peri rectal swab, 10 from blood, 4 from tracheal secretion, and one each from the following sources: tissue, prostatic secretion, throat swab, semen and CVP tip. The culture media for isolation of *S. aureus* were blood agar, mannitol salt agar and brain heart infusion (BHI) broth/agar.

***Identification of S. aureus Isolates: ***Identification of isolates was performed by Gram staining and routine biochemical tests including catalase, coagulase, mannitol salt fermentation, and DNase tests.

***Antibiotic Susceptibility Testing: ***Susceptibility testing was conducted by disk diffusion method according to the guidelines of Clinical and Laboratory Standards Institute (CLSI).^11^ Fusidic acid (susceptibility and resistance were ≥22 mm and <22 mm) and tigecycline susceptible breakpoints≥ 19 mm zone size were interpreted according to the European Committee on Antimicrobial Susceptibility Testing (EUCAST)and US FDA clinical breakpoints respectively. *S. aureus *ATCC 25923 was used as quality control strain.

***DNA Extraction: ***Extraction procedure was followed according to manufacturer’s instructions using Wizard Genomic DNA Extraction Kit (Promega Inc., Madison, USA).

***PCR Amplification and Detection of agr Groups: ***Primers provided by Integrated DNA Technologies (California, USA) were chosen from published sequences.^10^ The PCR assay was performed using green master mix (Promega Inc., Madison, USA). Amplified samples were analyzed by electrophoresis on a 1% agarose gel and stained with ethidium bromide.

***Statistical Analysis: ***Statistical analysis was performed using the software SPSS 17.0(SPSS Inc, Chicago, USA). Differences among different groups were analyzed using χ^2 ^test. *p* value less than 0.05 was considered as significant.

## RESULTS

Ninety clinical isolates were confirmed as *S. aureus *by Gram staining and standard biochemical tests. All isolates were mannitol fermenters and positive for catalase, DNase and coagulase. Resistance to oxacillin and cefoxitin or both according to CLSI presented the prevalence of MRSA to be 53.3 %.

***Prevalence and Association of MRSA and MSSA with Gender and Age: ***In our study, MRSA isolates were comparatively more prevalent in males 55.6% (35/63) than females 48.1% (13/27) whereas MSSA isolates were more prevalent in females 51.9% (14/27) than males 44.4% (28/63) with no significant statistical difference (*p* = 0.519).Age was categorized into three groups i.e., 1-18 years, 19-44 years and 44+ years. Higher prevalence 68.4% (13/19) and 60.9% (14/23) of MRSA was observed in age groups 44+ years and 1-18 years, respectively. Whereas, comparatively low prevalence (21/48) of MRSA was found in 19-44 years age group. Prevalence of MSSA was found to be 56.2% (27/48), 39.1% (9/23) and 31.6% (6/19) among groups aged 19-44, 1-18 and 44+ years, respectively. Association of MRSA and MSSA with age was found non-significant (*p *= 0.133).

***Antibiotic Resistance Profile of MRSA and MSSA Clinical Isolates:*** Table-I shows the antibiotic resistance of MRSA and MSSA isolates. Majority of MRSA isolates exhibited high level of resistance to penicillin, cefoxitin and fusidic acid and comparatively low resistance to vancomycin. MSSA isolates were also highly resistant to penicillin. Tigecycline against MRSA and vancomycin and cefoxitin against MSSA clinical isolates were found to be the most effective antibiotics.

**Fig.1:**
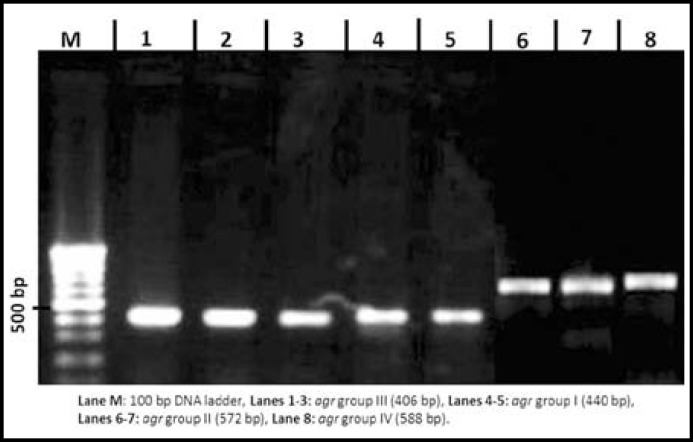
Analysis of PCR products for the identification of *agr* specific groups. Lane M: 100 bp DNA ladder. Lanes 1-3: PCR product of *agr* group III (406 bp). Lanes 4-5: PCR product of *agr* group I (440 bp). Lanes 6-7: PCR product of *agr* group II (572 bp). Lane 8: PCR product of *agr* group IV (588 bp).

***Prevalence of agr Specific Groups in MRSA and MSSA: ***Using multiplex PCR, MRSA and MSSA clinical isolates were grouped in four *agr* specific groups (Fig.1). Among all groups, *agr*I was the most prevalent followed by *agr*III and *agr*II. *agr*IV was absent in MRSA while 4.7% MSSA isolates were positive for it (Table-II).Non-type able *agr *group among MRSA and MSSA were 20.8% and 16.6%, respectively.

***Association between Antibiotic Resistance and agr Specific Groups: ***MRSA and MSSA isolates showed high resistance against penicillin with statistically significant difference (*p *= 0.002) in all *agr *groups as shown in Table-III. However, all *agr* containing MRSA and MSSA isolates were sensitive to tigecycline.

## DISCUSSION

In this study, prevalence of MRSA was found to be 53.3% which is consistent with the results reported by other researchers.^5^^,^^12^ Although higher rate of MRSA was observed in males with non-significant difference that is similar to the earlier reports.^2^^,^^3^

**Table-I:** Antibiotic resistance of MRSA and MSSA clinical isolates

	*Frequency (Resistance %)*		*Overall Resistance (%)*
*Antibiotic*	*MRSA*	*MSSA*	
Cefoxitin	45 (95.8)	0	51.1
Penicillin	47 (97.9)	37 (88.1)	93.3
Trimethoprim-sulfmethoxazole	11 (22.9)	16 (38.1)	30.0
Chloramphenicol	7 (14.6)	1 (2.4)	8.9
Erythromycin	17 (35.4)	9 (21.4)	28.9
Tetracycline	8 (16.7 )	3 (7.1)	12.2
Levofloxacin	9 (18.8)	4 (9.5)	14.4
Vancomycin	3 (6.3)	0	3.3
Linezolid	10 (20.8)	7 (21.4)	21.1
Fusidic acid	31 (64.6 )	10 (23.8)	45.5
Tigecycline	0	0	0
			

**Table-II:** Distribution of *agr* groups in MRSA and MSSA clinical isolates

*agr group*	*MRSA n (%)*	*MSSA n (%)*	*Total n (%)*
			
I	22 (45.8)	20(47.6)	42 (46.7)
II	2 (4.1)	4 (9.5)	6 (6.7)
III	14 (29.1)	9 (21.4)	23 (25.6)
IV	0	2 (4.7)	2 (2.2)
Non-typeable	10 (20.8)	7 (16.6)	17 (18.9)
Total	48 (100)	42 (100)	90 (100)

**Table-III:** Antibiotic resistance pattern of *S. aureus* isolates in *agr*-specific groups

	*agr-specific groups*						
*Antibiotic*	*agrI* *n (%)*	*agr II* *n (%)*	*agrIII* *n (%)*	*agrIV* *n (%)*	*Non-typeable* *n (%)*	*Total* *n (%)*	*p value*
Oxacillin	25 (59.5)	3 (50)	13 (56.5)	0	7 (41.1)	48 (53.3)	-
Cefoxitin	21 (50)	2 (33.3)	13 (56.2)	0	10 (58.2)	45 (50)	0.471
Penicillin	41 (97.6)	4 (66.6)	23 (100)	1 (50)	15 (88.2)	84 (93.3)	0.002
SXT*	11 (26.1)	1 (16.6)	10 (43.4)	1 (50)	4 (23.5)	27 (30)	0.477
Chloramphenicol	4 (9.5)	0	4 (17.3)	0	0	8 (8.9)	0.214
Erythromycin	12 (28.5)	2 (33.3)	7 (30.4)	1 (50)	4 (23.5)	26 (28.9)	0.635
Tetracycline	6 (14.2)	0	4 (17.3)	1 (50)	0	11 (12.2)	0.511
Levofloxacin	4 (9.5)	2 (33.3)	5 (21.7)	1 (50)	1 (5.8)	13 (14.4)	0.159
Vancomycin	0	0	2 (8.9)	0	1 (5.8)	3 (3.3)	0.390
Linezolid	9 (21.4)	1 (16.6)	4 (17.3)	1 (50)	4 (23.5)	19 (21.1)	0.857
Fusidic acid	20 (47.6)	2 (33.3)	11 (47.8)	1 (50)	7 (41.1)	41 (45.5)	0.960
Tigecycline	0	0	0	0	0	90( 0)	-

*SXT: Trimethoprim-sulfmethoxazole; n: No. of isolates.

Higher prevalence of MRSA was observed in case of patients aged 44+ years (68.4%) and 1-18 years (60.9%) which is close to the data,^3^ where it was 61.4% in 41-80 years age group. Similar results were reported in studies conducted in Malaysia and India.^13^^,^^14^

Previously, it has been shown that more cases of MRSA are reported from patients staying in intensive care units^2^^,^^3^and the same was observed in this study. Since the patients in ICUs are acutely ill and immune-compromised, it generates more risk for the infections.

A higher percentage (94%) of MRSA was found in nasal swabs than other specimens in contrast to the pus and sputum samples reported by others.^3^^,^^15^ This is due to the reason that nasal swabs constitute the major portion of samples coming from ICU.

For all the antibiotics tested, MRSA isolates showed great resistance than MSSA. Most of MRSA isolates presented multiple drug resistance. All isolates indicated 21.1% resistance to linezolid while MRSA presented 20.8% resistance. These results are in contradiction with other reports^3^^,^^16^ and up to 10% has been reported from Iran.^17^ This high level of resistance observed in isolates could be due to mutations of multiple gene copies, chloramphenicol-florfenicol resistance (*cfr*) gene carriage or misuse of this effective drug. Levofloxacin and tetracycline presented the resistance in the range of 16%-19% in MRSA isolates while 14.6% isolates were resistant to chloramphenicol which is similar to other studies.^17^^,^^18^

This study also identified 3% MRSA isolates which were resistant to vancomycin. This finding is in contrast with other studies from the region.^3^^,^^14^^,^^19^ Currently the drug of choice for treating life threatening infection caused by multidrug resistant MRSA is vancomycin so the emerging resistance against it would be a serious concern for the clinicians in Pakistan.

 In this study, tigecycline was the only drug with 100% sensitivity showing similarity with the earlier findings.^3^ Reports from other studies suggest that tigecycline is a good choice and has not yet been influenced by any of the resistance mechanisms which are involved in other antimicrobials.^19^^,^^20^

By amplification of the hyper-variable domain of the *agr* locus, we assigned *agr* groups to our clinical isolates. *Agr *group I was most prevalent group in both MRSA and MSSA clinical isolates followed by *agr* III which is similar with other studies.^10^^,^,*agr *group I and III are closely related having 80% sequence homology that would propose an exclusive genetic characteristics of our isolates and selection for the coexistence of *S*. *aureus *strains in the population.

No *agr *group was identified for 17 isolates which is in accordance with a previous report.^21^ Only 6 isolates were typed as *agr* II and 2 isolates as *agr* IV which represents reduced prevalence of these groups in our locality that is opposite to the earlier findings.^22^*agr* IV was only detected in case of MSSA while it was absent for MRSA isolates which is similar to results of few other studies.^10^^,^^21^ Their absence shows that competition does not favor these strains.

Although *agr* I was dominant in all sources, hospital wards and age groups, it was higher in nasal swabs (50%), OPD (50%) and 44+ years age group (57.8%) respectively. *agr*III was more prevalent in sources other than pus or nasal swabs (31.8%), in 44+ years age group (10.4%) as compared to other age groups and in outpatients (28.2%) as compared to hospitalized patients. Resistance profile suggests that *agr* III isolates are more resistant than *agr* I. Resistance to oxacillin is almost similar in all *agr* groups except *agr* IV and all *agr* IV isolates were resistant to penicillin in this study similar to the report by other study.^22^

In conclusion, *agr*I was the most prevalent group in all the hospital departments, all type of sources and age groups followed by *agr*III. The uniform fitness of *S*. *aureusagr *groups in some cases suggests that they also have comparable competitive ability within the host. The allocation of *agr* groups in this study perhaps reflects ecological and geographical structuring or sampling bias.

## Notes

***Source of funding: ***Higher Education Commission, Pakistan.
